# Supplementary material for: Quality Assessment of HDR/WCG Images Using HDR Uniform Color Spaces
Source: J Imaging. 2019 Jan 14;5(1):18. doi: 10.3390/jimaging5010018 (PMC8320861; doi:10.3390/jimaging5010018)
Supplement: Supplementary file 1 [file jimaging-05-00018-s001.zip › jimaging-390731-SI.pdf]

Supplementary Materials: Quality assessment of HDR/WCG images using HDR uniform color spaces.

Maxime Rousselot [1], Olivier Le Meur [2]*, Rémi Cozot [2] and Xavier Ducloux [1]

In this supplementary materials, we present in details the SDR metrics selected in the article: **Quality assessment of HDR/WCG images using HDR uniform color spaces.**

1. PSNR

The peak Signal-to-Noise Ratio (PSNR) is a simple metric that evaluates the distortion between a reference and a distorted image.

$$\mathrm{PSNR} = 10 \log_{10}\left(\frac{\mathrm{DR}^2}{\mathrm{MSE}(X,Y)}\right) \tag{1}$$

where DR is traditionally the range of the possible luminance values (255 for an 8 bits representation). The $\mathrm{MSE}(X,Y)$ corresponds to the mean square error between the reference and the distorted images:

$$\mathrm{MSE} = \frac{1}{IJ}\sum_i \sum_j [X(i,j) - Y(i,j)]^2 \tag{2}$$

where I and J are the image resolution and i and j are the pixels spatial coordinates of the X and Y images.

2. S-CIELab

The S-CIELab [1] metric is the spatial extension of the CIE-*Lab* color space. Indeed, the color difference metric $\Delta \mathrm{E}_{ab}$ is not correlated with the perception of differences in natural images but in large uniform patches. The goal of this extension is to take into account the blurring effect of the HVS. To achieve that, the images in the XYZ color space are transformed into an opponent color space as follows:

$$\begin{bmatrix} O_1 \\ O_2 \\ O_3 \end{bmatrix} = \begin{bmatrix} 0.279 & 0.72 & -0.107 \\ -0.449 & 0.29 & -0.077 \\ 0.086 & -0.59 & 0.501 \end{bmatrix} \begin{bmatrix} X \\ Y \\ Z \end{bmatrix} \tag{3}$$

Each component O is then filtered, using filters that approximate the Contrast Sensitivity Function (CSF) of the HVS. This is accomplished using filters with two-dimensional separable convolution kernels f of the form:

$$f(x,y) = k \sum_i w_i E_i(x,y) \tag{4}$$

where

$$E_i = k_i \exp[-(x^2 + y^2)/\sigma_i^2] \tag{5}$$

The parameters for w_i and σ_i are different for each component O_1, O_2, O_3, the scale factor k_i is chosen so that each E_i sums to 1. The scale factor k is chosen so that for each color plane, the kernel f sums to 1. Then, the filtered images are converted back to the XYZ color space from which the uniform color space Lab can be calculated. Once this transformation is done on the reference image and on the distorted image, a color difference map using the color difference metric, ΔE, can be calculated. The new distortion map is called ΔE^S. To obtain a unique value estimating the overall perceptual difference between two images, the average of this map is computed:

$$\overline{\Delta \mathrm{E}^S} = \frac{1}{IJ}\sum_i^I \sum_j^J \Delta \mathrm{E}^S(i,j) \tag{6}$$

The S-CIELab metric was originally designed to measure color reproduction errors of printed digital images. It is also often used as an image quality metric because of its simple implementation.

3. SSIM

3.1. Color-blind SSIM:

The structural similarity index (SSIM) [2] is based on the assumption that the HVS is highly adapted to extract the structure of natural scene. SSIM is composed of the comparison of three characteristics calculated from the luminance component of two images X and Y, one being the reference image and the other one the distorted image.

- First, the luminance of each signal is compared using the $l(X,Y)$ function:

$$l(X,Y) = \frac{2\mu_X\mu_Y + C_1}{\mu_X^2 + \mu_Y^2 + C_1} \tag{7}$$

- Then, the $c(X,Y)$ function which represents the contrast comparison is calculated :

$$c(X,Y) = \frac{2\sigma_X\sigma_Y + C_2}{\sigma_X^2 + \sigma_Y^2 + C_2} \tag{8}$$

- Finally, the structure of the images is compared using the function $s(X,Y)$:

$$s(X,Y) = \frac{\sigma_{XY} + C_3}{\sigma_X\sigma_Y + C_3} \tag{9}$$

where μ_X and μ_Y are the means of the images X and Y, σ_X and σ_Y are their standard deviations and σ_{XY} is the covariance between them. C_1, C_2 and C_3 are used to prevent the division by zero. These three components are combined as follows:

$$\text{SSIM}(X,Y) = [l(X,Y)^\alpha \times c(X,Y)^\beta \times s(X,Y)^\gamma] \tag{10}$$

Usually the weights α, β and γ are set to 1. The SSIM is often calculated locally and not globally (like in previous equation) allowing to have more detailed information about the distortion. The SSIM is then calculated for each pixel through a sliding window. In [2], Wang et al. used a sliding window in which values are weighted using an 11×11 circular-symmetric Gaussian weighting function $W = \{w_n | n = 1, 2, ..., N\}$. The statistics are then calculated for each pixel x of X and y of Y:

$$\mu_x = \sum_{n=1}^{N} w_n x_n \tag{11}$$

$$\sigma_x = (\sum_{n=1}^{N} w_n (x_n - \mu_x)^2)^{\frac{1}{2}} \tag{12}$$

$$\sigma_{xy} = \sum_{n=1}^{N} w_n (x_n - \mu_x)(y_n - \mu_y) \tag{13}$$

A SSIM index is calculated for each pixel and its associated window using the equation (10). To obtain an overall quality measure of the image, the mean of the SSIM indexes is calculated:

$$\text{MSSIM}(X,Y) = \frac{1}{IJ} \sum_{i=1}^{I} \sum_{j=1}^{J} \text{SSIM}(x_{ij}, y_{ij}) \tag{14}$$

where I and J represent the image resolution.

We keep the name SSIM instead of MSSIM to avoid confusion with the multiscale SSIM (MS-SSIM) (see section 4).

3.2. SSIM for color images (SSIMc):

The SSIM was only defined for luminance information. In [3], Wang et al. extended the SSIM to video as well as to chrominance information. They used SSIM for each component of the video sequence encoded in the Y'CrCb [4] color space. The SSIM scores for the three components are then aggregated using a weighted arithmetic mean:

$$\text{SSIMc}(i,j) = 0.8\ \text{SSIM}_{Y'}(i,j) + 0.1\ SSIM_{Cr}(i,j) + 0.1\ \text{SSIM}_{Cb}(i,j) \tag{15}$$

where i and j are the pixel coordinates of the SSIM map. As for the color-blind SSIM metric, an overall quality score is obtained by averaging all SSIMc scores.

3.3. CSSIM

Another solution to include chrominance information in SSIM was proposed in [5]. Authors proposed to add a new comparison that use a S-CIELab ΔE^S_{ab} distortion map (see section 2) and is called the color comparison. As for precedent comparison, the color comparison is calculated for each pixel x of the reference image X and each pixel y of the distorted image Y:

$$c_r(x,y) = 1 - \frac{1}{k} \times \Delta E^S_{ab}(x,y) \tag{16}$$

k is a constant equal to 45. The SSIM index (cf. Equation (10)) is then adapted as follows:

$$\text{CSSIM} = \frac{1}{IJ}\sum_i^I\sum_j^J l(x_{ij},y_{ij}) \times c(x_{ij},y_{ij}) \times s(x_{ij},y_{ij}) \times c_r(x_{ij},y_{ij}) \tag{17}$$

4. Multiscale-SSIM (MS-SSIM)

Wang et al. [6] improved the SSIM index by using a multiscale approach (MS-SSIM). They proposed to apply the comparison functions used in SSIM at different scales of the images. The goal is to incorporate image details at different resolutions. The different scales are obtained after a low-pass filtering and a downsampling of the images. The index of the original scale is 1 and the index of the highest scale is M. The luminance comparison (equation (14)) is only done at the highest scale. The multiscale SSIM evaluation is obtained using the following combination:

$$\text{MS-SSIM} = [L_M(X,Y)]^{\alpha_M}.\prod_{m=1}^{M}[C_m(X,Y)]^{\beta_m}[S_m(X,Y)]^{\gamma_m} \tag{18}$$

Where X and Y are the reference and the distorted images, L, C and S are the mean of the pixelwise luminance, contrast and structure comparison functions l, c and s defined in section 3 for the different image scales m. α, β and γ are the parameters of the metric.

In [6], the authors proposed a 5 scales MS-SSIM where the parameters are : $\beta_1 = \gamma_1 = 0.0448$, $\beta_2 = \gamma_2 = 0.2856$, $\beta_3 = \gamma_3 = 0.3001$, $\beta_4 = \gamma_4 = 0.2363$ and $\alpha_5 = \beta_5 = \gamma_5 = 0.1333$.

5. FSIM

5.1. Color-blind FSIM

The feature similarity (FSIM) index [7] allows to overcome a limit of SSIM and MS-SSIM: in these algorithms, a simple average is used to pool the SSIM local distortion map (equation (14)), each pixel

having then the same importance. The authors of FSIM made the assumption that different locations can have very different contributions to the quality perception of an image.

FSIM extracts from the image two different features: the phase congruency and the gradient magnitude.

The phase congruency model postulates that details of an image become visible where the Fourier components are maximal in phase. The phase congruency can be considered as a measure of the significance of local structure. To compute this feature, the authors used the method developed by Kovesi [8]. The comparison of the phase congruency planes of the reference image ($PC_X(i,j)$) and of the distorted image ($PC_Y(i,j)$), i and j being the pixels spatial coordinates, is done as follows:

$$S_{PC}(i,j) = \frac{2PC_X(i,j) \times PC_Y(i,j) + T_1}{PC_X^2(i,j) + PC_Y^2(i,j) + T_1} \tag{19}$$

The feature called gradient magnitude is calculated to take into account contrast information (the phase congruency is contrast invariant). The comparison of the gradient magnitude planes of the reference image ($G_X(i,j)$) and the distorted image ($G_Y(i,j)$) is done as follows:

$$S_G(i,j) = \frac{2G_X(i,j) \times G_Y(i,j) + T_2}{G_X^2(i,j) + G_Y^2(i,j) + T_2} \tag{20}$$

Finally, the FSIM is calculated as follows:

$$S_L(i,j) = [S_{PC}(i,j)] \times [S_G(i,j)] \tag{21}$$

$$PC_m(i,j) = \max(PC_X(i,j), PC_Y(i,j)) \tag{22}$$

$$\text{FSIM} = \frac{\sum_i \sum_j S_L(i,j) PC_m(i,j)}{\sum_i \sum_j PC_m(i,j)} \tag{23}$$

The parameters values of FSIM are T_1=0.85 and T_2=160

5.2. FSIM for color images (FSIMc):

To add chrominance consideration in FSIM, the authors of the metric proposed the FSIMc index [7]. Images are first converted from the *RGB* (BT.709) color space to the YIQ color space [9] :

$$\begin{bmatrix} Y \\ I \\ Q \end{bmatrix} = \begin{bmatrix} 0.299 & 0.587 & 0.114 \\ 0.596 & -0.274 & 0.322 \\ 0.211 & -0.523 & 0.312 \end{bmatrix} \begin{bmatrix} R \\ G \\ B \end{bmatrix} \tag{24}$$

and then a comparison between the chrominance components (I_X and I_Y and respectively Q_X and Q_Y) performed for every pixel (*i,j*):

$$S_I(i,j) = \frac{2I_X(i,j) \times I_Y(i,j) + T_3}{I_X^2(i,j) + I_Y^2(i,j) + T_3} \tag{25}$$

$$S_Q(i,j) = \frac{2Q_X(i,j) \times Q_Y(i,j) + T_4}{Q_X^2(i,j) + Q_Y^2(i,j) + T_4} \tag{26}$$

The authors incorporate these comparisons straightforwardly into the FSIM index:

$$\text{FSIMc} = \frac{\sum_i \sum_j S_L(i,j) PC_m(i,j) \times [S_I(i,j) \times S_Q(i,j)]^\lambda}{\sum_i \sum_j PC_m(i,j)} \tag{27}$$

The values of FSIMc parameters are T_3=200, T_4=200 and λ=0.03.

6. PSNR-HVS and PSNR-HVS-M

PSNR-HVS [10] is a modified version of the PSNR. It takes into account the contrast sensitivity function (CSF) which measures the human sensibility to spatial frequencies.

The PSNR-HVS is calculated as follows:

$$\text{PSNR-HVS} = 10\log_{10}\left(\frac{255^2}{\text{MSE}_H}\right) \tag{28}$$

The MSE_H is calculated as follows:

$$\text{MSE}_H = K\sum_{i=1}^{I-7}\sum_{j=1}^{J-7}\sum_{m=1}^{8}\sum_{n=1}^{8}((X[m,n]_{ij} - Y[m,n]_{ij})T_c[m,n])^2, \tag{29}$$

I and J are the images size. X_{ij} and Y_{ij} are the 8×8 DCT coefficients of the reference and distorted images for which the coordinates of the left upper corner are i and j. T_c is the matrix of correcting factors that takes into account the HVS spatial sensitivity. m and n are the coordinates of the DCT block coefficients. Finally, K is equal to $1/[(I-7)(J-7)64]$.

In [11], Ponomarenko et al. improved the performances of PSNR-HVS by adding a model of the contrast masking: the fact that any DCT coefficient X_{ij} of a block can mask any other block coefficients except the DC coefficient (the mean luminance). This metric is called PSNR-HVS-M.

To estimate the masking effect, a weighted energy E_w is calculated for each DCT block X:

$$E_w(X) = \sum_{m=1}^{8}\sum_{n=1}^{8} X[m,n]^2 C[m,n], \tag{30}$$

where $C[m,n]$ is a matrix of correcting factors that models the contrast masking. The value of the masking effect can be too high if an image block belongs to an edge. To overcome this effect, a correction is then applied on E_w.

$$E_m(D) = E_w(D)\delta(D)/16 \tag{31}$$

The correcting factor $\delta(D)$ is calculated using the local variance V of the four 4×4 sub-block $D1$, $D2$, $D3$ and $D4$ of the 8×8 image DCT block D.

$$\delta(D) = (V(D1) + V(D2) + V(D3) + V(D4))/4V(D) \tag{32}$$

The masking model is then applied on the distorted ($Y[m,n]$) and reference ($X[m,n]$) images DCT blocks to obtain visible distortion blocks ΔXY as follows:

$$\Delta XY[m,n] = \begin{cases} X[m,n] - Y[m,n] & \text{if} \quad m = 0, n = 0 \\ 0 & \text{if} \quad \mid X[m,n] - Y[m,n] \mid \leq E_{norm}/C[m,n] \\ X[m,n] - Y[m,n] - E_{norm}/C[m,n] & \text{if} \quad X[m,n] - Y[m,n] > E_{norm}/C[m,n] \\ X[m,n] - Y[m,n] - E_{norm}/C[m,n] & \quad \text{otherwise} \end{cases} \tag{33}$$

where

$$E_{norm} = \sqrt{\max(E_m(X), E_m(Y))/64} \tag{34}$$

The new MSE is then calculated as in equation (29):

$$\text{MSE}_{HVSM} = K\sum_{i=1}^{I-7}\sum_{j=1}^{J-7}\sum_{m=1}^{8}\sum_{n=1}^{8}((\Delta XY_{ij}[m,n])T_c[m,n])^2 \tag{35}$$

The PSNR-HVS-M is then calculated as in equation (28):

$$\text{PSNR-HVS-M} = 10\log_{10}\left(\frac{255^2}{\text{MSE}_{HVSM}}\right) \tag{36}$$

7. PSNR-HMA

7.1. Color-blind PSNR-HMA

PSNR-HMA [12] is an improvement of PSNR-HVS-M. PSNR-HVS-M has poor performances when used on images that contain contrast change and mean shift. PSNR-HMA was created to take into account the particularities of the HVS concerning these two distortions.

For a given reference image X and a given distorted image Y and their means $\overline{X}$ and $\overline{Y}$, a new image C is calculated :

$$C = Y + Delt \tag{37}$$

$$Delt = \overline{X} - \overline{Y} \tag{38}$$

The correcting factor $Popr$ is computed to assess possible contrast change :

$$Popr = \frac{\sum(X - \overline{X})(C - \overline{C})}{\sum(C - \overline{C})} \tag{39}$$

The new image D is calculated:

$$D = (C - \overline{C}).Popr + \overline{C} \tag{40}$$

This is the image that minimizes the Mean square error with A. Two MSE_{HVSM} are calculated (Equation (35)) :

$$M_1 = \text{MSE}_{HVSM}(X, C) \tag{41}$$

$$M_2 = \text{MSE}_{HVSM}(X, D) \tag{42}$$

If $M_1 > M_2$, the contrast change needs to be dealt with as follows:

$$M_1 = M_2 + \begin{cases} (M_1 - M_2)C_1 & \text{if} \quad Popr < 1 \\ (M_1 - M_2)C_2 & \text{if} \quad Popr \geq 1 \end{cases} \tag{43}$$

Then the final MSE_{HMA} is calculated as follows:

$$\text{MSE}_{HMA} = M_1 + Delt^2.C_3 \tag{44}$$

The values of the metric constants are: C_1=0.002, C_2=0.25 and C_3= 0.04

7.2. PSNR-HMA for color images (PSNR-HMAc):

To include the chrominance components into the metric, Ponomarenko et al. [12] proposed to aggregate the MSE_{HMA} for each channel using the $Y'CrCb$ color space [4] as follows:

$$\text{MSE}_{HMA} = (\text{MSE}_{HMA}(Y) + C_4 \times \text{MSE}_{HMA}(Cr) + C_4 \times \text{MSE}_{HMA}(Cb))/(1 + 2 \times C_4) \tag{45}$$

The values of the metric new constant is C_4=0.5.

References

1. Zhang, X.; Wandell, B.A. A spatial extension of CIELAB for digital color-image reproduction. *Journal of the Society for Information Display*, 5, 61–63.

2. Wang, Z.; Bovik, A.C.; Sheikh, H.R.; Simoncelli, E.P. Image quality assessment: from error visibility to structural similarity. *IEEE Transactions on Image Processing* **2004**, *13*, 600–612. doi:10.1109/TIP.2003.819861.
3. Wang, Z.; Lu, L.; Bovik, A.C. Video quality assessment based on structural distortion measurement. *Signal Processing: Image Communication* **2004**, *19*, 121 – 132. doi:10.1016/S0923-5965(03)00076-6.
4. Parameter values for the HDTV standard for production and international program exchange. Rec BT.709-6, ITU-R, 2015.
5. Hassan, M.A.; Bashraheel, M.S. Color-based structural similarity image quality assessment. 2017 8th International Conference on Information Technology (ICIT), 2017, pp. 691–696. doi:10.1109/ICITECH.2017.8079929.
6. Wang, Z.; Simoncelli, E.P.; Bovik, A.C. Multiscale structural similarity for image quality assessment. The Thrity-Seventh Asilomar Conference on Signals, Systems Computers, 2003, Vol. 2, pp. 1398–1402. doi:10.1109/ACSSC.2003.1292216.
7. Zhang, L.; Zhang, L.; Mou, X.; Zhang, D. FSIM: A Feature Similarity Index for Image Quality Assessment. *IEEE Transactions on Image Processing* **2011**, *20*, 2378–2386. doi:10.1109/TIP.2011.2109730.
8. Kovesi, P. Image features from phase congruency. *Videre: Journal of computer vision research* **1999**, *1*, 1–26.
9. Yang, C.C.; Kwok, S.H. Efficient gamut clipping for color image processing using LHS and YIQ. *Optical Engineering* **2003**.
10. Egiazarian, K.; Astola, J.; Ponomarenko, N.; Lukin, V.; Battisti, F.; Carli, M. New full-reference quality metrics based on HVS. Proceedings of the Second International Workshop on Video Processing and Quality Metrics, 2006, Vol. 4.
11. Ponomarenko, N.; Silvestri, F.; Egiazarian, K.; Carli, M.; Astola, J.; Lukin, V. On between-coefficient contrast masking of DCT basis functions. Proceedings of the third international workshop on video processing and quality metrics, 2007, Vol. 4.
12. Ponomarenko, N.; Ieremeiev, O.; Lukin, V.; Egiazarian, K.; Carli, M. Modified image visual quality metrics for contrast change and mean shift accounting. CAD Systems in Microelectronics (CADSM), 2011 11th International Conference The Experience of Designing and Application of. IEEE, 2011.
